# Chemical and Enzymatic Fiber Modification to Enhance the Mechanical Properties of CMC Composite Films

**DOI:** 10.3390/polym14194127

**Published:** 2022-10-02

**Authors:** Xiaobao Li, Zhengjie Tang, Zhenbing Sun, John Simonsen, Zhinan Luo, Xiaoping Li, Jeffery J. Morrell

**Affiliations:** 1 Yunnan Key Laboratory of Wood Adhesives and Glue Products, Southwest Forestry University, Kunming 650224, China; 2 Department of Wood Science and Engineering, Oregon State University, Corvallis, OR 97331, USA; 3 International Joint Research Center for Biomass Materials, Southwest Forestry University, Kunming 650224, China; 4 National Centre for Timber Durability and Design Life, University of the Sunshine Coast, Brisbane, QLD 4102, Australia

**Keywords:** eucalyptus bark, Yunnan pine wood, bamboo culms, industrial hemp hurd, FTIR, XRD, TG, mechanical properties

## Abstract

Carboxymethyl cellulose (CMC) is a cellulose derivative that can be obtained from wood, bamboo, rattan, straw, and other cellulosic materials. CMC can be used to produce biofilms for many purposes, but the properties of these resulting films make them unsuitable for some applications. The effects of three kinds of plant fiber addition on CMC film properties was investigated using CMC derived from eucalyptus bark cellulose. Tensile strength (TS) and elongation at break (EB) of CMC/sodium alginate/glycerol composite films were 26.2 MPa and 7.35%, respectively. Tensile strength of CMC composite films substantially increased, reaching an optimum at 0.50 g of fiber. The enhancement due to industrial hemp hurd fiber on CMC composite films was more obvious. Pretreatment with hydrogen peroxide (H_2_O_2_) and glacial acetic acid (CH_3_COOH) produced films with a TS of 35.9 MPa and an EB of 1.61%. TS values with pectinase pretreated fiber films was 41.3 MPa and EB was 1.76%. TS of films pretreated with pectinase and hemicellulase was 45.2 MPa and EB was 4.18%. Chemical and enzymatic treatment both improved fiber crystallinity, but film tensile strength was improved to a greater extent by enzymatic treatment. Surface roughness and pyrolysis residue of the film increased after fiber addition, but Fourier transform infrared spectroscopy (FTIR), opacity, and water vapor transmission coefficients were largely unchanged. Adding fiber improved tensile strength of CMC/sodium alginate/glycerol composite films and broadened the application range of CMC composite films without adversely affecting film performance.

## 1. Introduction

Petroleum-based composite films are widely used in the food, pharmaceutical, and chemical industries due to their good properties and low cost. However, there is increasing interest in moving away from fossil fuel-based materials to renewable natural polymers such as cellulose. Cellulose can be modified to produce carboxymethyl cellulose (CMC), an odorless, tasteless, non-toxic, neutral or slightly alkaline, white or yellowish powder. CMC is hygroscopic, relatively light and heat stable, and transparent in aqueous solutions [1]. CMC is widely used in oil drilling, food packaging, concrete modification, and soil improvement [2,3,4,5,6,7]. The degree of substitution (DS, average number of hydroxyl groups substituted with carboxymethyl groups per anhydroglucose unit (AGU)) has a major influence on the properties, and therefore the potential uses, of CMC. The theoretical maximum DS of CMC is three; the degree of substitution of CMC directly affects the solubility, emulsification, thickening, stability, acid resistance, and salt resistance of CMC [8]. CMC with a super high degree of substitution (DS = 1.7~3.0) is often used in the textile, printing, and dyeing industry. CMC with a high degree of substitution (DS = 1.0~1.2) is often used as a food additive. CMC (DS = 0.6~0.9) with a low degree of substitution is commonly used in industrial drilling, ceramics, detergents, building materials, etc. [9]. In past research reports, some rich and underutilized plant cellulose sources have been used as raw materials for CMC production to replace cellulose materials obtained from cotton linter or wood bleached pulp. However, there is little information about CMC from eucalyptus bark cellulose. Eucalyptus bark comes from the eucalyptus tree, fallen off every year and is rich in cellulose. Eucalyptus wood is widely used in wood-based panel manufacturing, pulp and paper, etc., but the bark is a by-product of eucalyptus that typically has no use [10].

CMC also has great potential to create degradable films. However, single CMC composites are not suitable for all applications due to their poor mechanical properties and water permeability. Thickeners such as starch, sodium alginate, or gelatin can improve the mechanical properties and reduce moisture absorption of CMC films. When the ratio of CMC to corn starch is 4:6, the tensile strength of single starch film can be significantly improved, from 3.8 to 17.0 MPa [11], whereas addition of 1.5% sodium alginate to a CMC/chitosan mixture produced antibacterial food packaging films with tensile strengths and elongation at break of 65.32 MPa and 17.85%, respectively [12]. Adding 3.2% gelatin to a 0.8% CMC solution, the tensile strength of CMC composite film became 7.84 ± 0.30 MPa [13].Therefore, sodium alginate is often used as a thickener in CMC composite films. Plasticizers such as sorbitol, polyethylene glycol, or glycerin help improve ductility and tensile strength of composite samples. After 1.2% sorbitol was blended with CMC–gelatin–chitosan as plasticizer, the elongation at break of the composite film became 9.23% [14]. Adding 25% (*w*/*w*) glycerol to starch–alginate–CMC increased elongation at break by 58.6% [15]; 5 wt% polyethylene glycol blended with clay minerals–CMC, the maximum elongation at break was only 8.0% [16]. This comparison shows that the plasticizing effect of glycerin is the best.

CMC composite film properties can also be enhanced via addition of fibers derived from a variety of natural sources. For example, tensile strength increased 1.93 times with the addition of 8% sugar cane fiber to a polyvinyl alcohol (PVA) composite film [17]. The addition of wheat bran fiber to a corn starch composite film was associated with an increase in tensile strength from 2 to 5.07 MPa and a decrease in elongation at break from 60 to 28% [18]. The addition of cassava bagasse fiber to a cassava starch/glycerol composite film significantly increased maximum tensile strength (from 1.23 ± 0.15 to 7.78 ± 0.83 MPa) [19].The ability of small amounts of fiber to enhance film properties have seen these products used in construction, automotive, packaging, sports, and biomedicine. These applications highlight the potential for adding fibers to improve the properties of CMC films. However, there is little information about the effects of fiber-separation methods on the properties of CMC film. Fiber can be separated by mechanical or chemical methods. The mechanical processing method often results in ripped or torn fibers with a high elastic modulus and elongation at break, but poor tensile strength [20]. Chemical methods include nitric acid + potassium chlorate (HNO_3_ + KClO_3_), sodium hypochlorite (NaClO), hydrogen peroxide + glacial acetic acid (H_2_O_2_ + HAc), and sodium hydroxide (NaOH); sulfate can represent a gentler separation method [21]. For example, fibers prepared by H_2_O_2_ + HAc were not hollow, and resulted in separated whole fibers with high tensile strength. There have been few reports on enzymatic separation of plant fibers; however, enzymes have catalytic efficiencies that are 10^7^–10^13^ times higher than non-enzymatic catalysts [22,23]. Enzymatic catalytic reactions are substrate-specific substrates, do not affect other raw materials, and cause less damage to raw materials. As enzymes are proteins, they are biodegradable, more environmentally friendly, and are a better choice for fiber separation [20,24,25].

In this study, chemical and enzymatic methods were used to separate plant fibers from different raw materials. The fibers were characterized and the properties of fiber-amended CMC composites were studied. We also suggest a method for making CMC films of high quality and improving the value of eucalyptus bark.

## 2. Materials and Methods

### 2.1. Materials

Eucalyptus bark (Eucalyptus globulus Labill.), Yunnan pine wood (Pinus yunnanensis), bamboo culms (Neosinocalamus affinis), and industrial hemp hurd (Cannabis sativa) were all obtained locally (Kunming, Yunnan Province, China). They were ground to pass through a 40–60 mesh screen (250–420 μm) before cellulose extraction and chemical analysis. The contents of benzene-alcohol extract, holo-cellulose, cellulose, hemicellulose, and lignin were determined according to Chinese Standards GB/T 2677.6-1994 (Determination of organic solvent extract in paper raw materials), GB/T 2677.10-1995 (Determination of holo-cellulose in paper raw materials), GB/T 744-1989 (Determination of α-cellulose in pulp), and GB/T 2677.8-94 (Determination of acid-insoluble lignin in paper raw materials).

### 2.2. Plant Fiber Separation

Fibers of Yunnan pine wood, industrial hemp hurd, and bamboo culms were cut into pieces 3–5 by 3–5 by 5–10 mm (width by thickness by length) prior to chemical and enzymatic fiber extraction.

For chemical fiber separation: five grams of Yunnan pine wood, Bamboo culms, or industrial hemp hurd were placed in beakers and immersed in 100 mL of a 50:50 mixture of hydrogen peroxide (H_2_O_2_): glacial acetic acid (CH_3_COOH) at 70 °C until the sample turned white. The samples were washed with distilled water until the pH was 7, then the samples were shaken slightly to separate the fibers. These procedures were performed in triplicate.

Enzymatic fiber separation: three enzymatic methods were used to obtain fibers. In the first method, five grams of a given material was treated with 100 mL of 5.00% lipase solution at 50 °C, stirred (900–1000 r/min) for 3 h, then the solution was filtered off and the residual materials were treated with GB2677.10-1995 (Determination of the content of holo-cellulose from paper raw materials) to remove most of the lignin. The residual material was immersed in 40 mL of 50 °C distilled water and stirred (900–1000 r/min) for more than 3 h until most of the fibers were separated, then washed with distilled water to obtain residues. The second method used the same procedure, but then immersed the fibers in 40 mL of 5% pectinase solution instead of distilled water. The final method used the same procedure, but then immersed the materials in 40 mL of 5% a 20:20 mixture of 5% pectinase solution and 5% hemicellulase solution instead of distilled water. Lipase (CAS:9001-62-1), pectinase (CAS:9032-75-1), and hemicellulose (CAS:9025-56-3) were obtained from Aladdin Biotechnology Co., Ltd., (Shanghai, China) and had enzyme activities of 100,000; 30,000; and 5000 U/g, respectively. Each fiber material/enzymatic treatment combination was prepared in triplicate.

### 2.3. Preparation of Cellulose, CMC, and CMC Composite Films

Four grams of cellulose extracted from eucalyptus bark according to Chinese Standard GB/T744-1989 (Determination of α-cellulose from pulp) was mixed with 80 mL of 100% ethanol(CH_3_CH_2_OH) and 20 mL of 30% NaOH solution and then stirred(900–1000 r/min) for 60 min at 30 °C. Five grams of sodium chloroacetate (C_2_H_2_ClNaO_2_) were added and heated at 65 °C for 3 h. The sample was washed with 90% glacial acetic acid to a pH of 7, then washed with 80% ethanol 3 times and 95% ethanol once, before being oven-dried at 65 °C for 3 h to obtain CMC.

**Figure 1:**
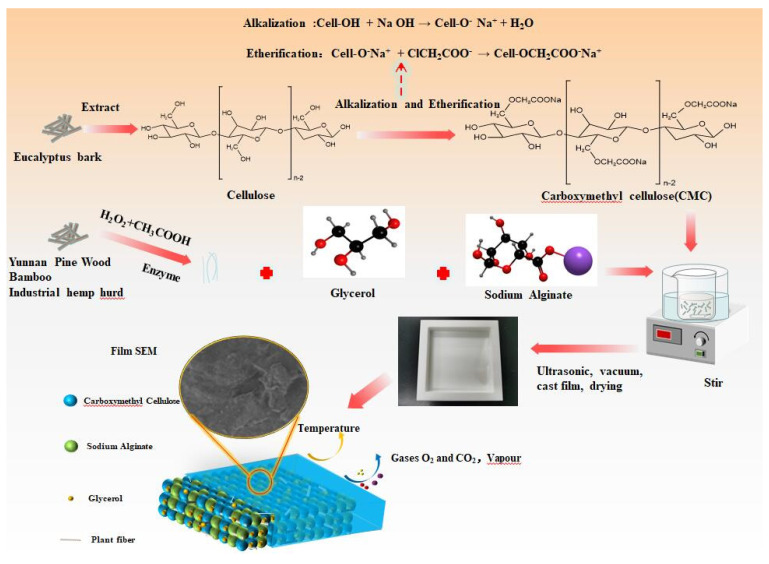
Preparation of CMC composite films.

One gram of CMC was mixed with 0.40 g sodium alginate (C_6_H_7_NaO_6_), 0.15 g glycerol (C_3_H_8_O_3_), and 49.00 g distilled water and stirred (900–1000 r/min) at 70 °C for 15 min. The mixture was treated in an ultrasonic bath for 10 min and vacuumed for 45 min to remove air bubbles. The solution was poured into a polytetrafluoroethylene (PTFE) mold and cured at 30 °C for 48 h to obtain CMC composite films. The fiber-modified CMC composite film process was similar except that 0.1, 0.3, and 0.5 g of a given plant fiber was added during mixing (shown in Figure 1).

### 2.4. Fiber and Composite Film Characterization

**Fiber dimensions:** The length and width of 100 fibers, as well as the cell wall thickness and cell cavity width of each of the chemically or enzymatically prepared fibers were measured under a light microscope by ImageJ software.

**Sample microstructure:** The microstructure of eucalyptus bark powder, cellulose, CMC, and CMC composite films were observed by placing a sample on an aluminium stub and coating with gold/palladium before observation with the Czech TESCAN MIRA LMS field emission scanning electron microscope at an accelerating voltage of 200 eV to 30 KeV. A minimum of five fields were examined per material.

**Fourier-transform infrared spectroscopy (FTIR):** Eucalyptus bark powder, cellulose, and CMC were mixed with KBr and formed into a pellet while the CMC composite films were directly analyzed on a Nicolet i50 FTIR analyzer (Thermo Nicolet Corporation, Madison, WI, USA) with a scanning range of 500 to 4000 cm^−1^ and 64 scans. Baseline correction was performed to analyze the spectral differences between plant fibers obtained by different treatments.

**X-ray diffractometer analysis (XRD):** The crystal structures of eucalyptus bark, cellulose, and the CMC films were studied by X-ray diffraction (XRD) on an Ultima IV X-ray diffractometer (Rigaku Corporation, Tokyo, Japan) using a scanning angle from 5 to 60°, a step size of 0.026° (accelerating current = 30 mA and voltage = 40 kV), and Cu-Kα radiation of *λ* = 0.154 nm. The degree of crystallinity (DOC, %) was calculated according to the formula:

(1) $$\mathrm{DOC}\%=\frac{I_{Max}-I_{Am}}{I_{Max}}\times 100$$

$I_{Max}$ is the maximum intensity of the main peak (about 22°), and $I_{Am}$ is the diffraction intensity of amorphous cellulose (about 15°).

**Thermogravimetric (TG) analysis:** Approximately 5 to 6 mg of sample powder ground to pass through an 80 to 120 mesh screen and placed into sample holders for analysis on a TGA92 thermo gravimetric analyzer (KEP Technologies EMEA, Caluire, France). N_2_ was used as the shielding gas and Al_2_O_3_ as the reference compound. The temperature was increased from 35 to 800 °C at a rate of 20 °C/min to generate a thermogravimetric curve.

**Degree substitution (DS) of CMC:** The degree of substitution of hydroxyl groups has an important influence on resulting CMC properties. The degree of substitution was determined by the acidimeter method by weighing 0.2 g (accuracy 0.1 mg) of the sample, dissolving it in 80 mL of water, stirring for 10 min, and adjusting the pH to 8.0. The sample was titrated using sulphuric acid (H_2_SO_4_) with continuous stirring to pH 3.74. The volume (mL) of sulphuric acid titration solution used was recorded (to the nearest 0.05 mL). The degree of substitution (*DS*) was then calculated using the amount required to reach the end point according to Equations (1) and (2), as follows (Table 1).

(2) $$B=\frac{2cV}{m}$$

(3) $$DS=\frac{0.162B}{1-0.08B}$$

where *B* = amount of carboxymethyl substance contained in the sample, mmol/g;

*m* = quality of the sample, g;

*c* = concentration of sulphuric acid standard titration solution, mol/L;

*V*= volume value of standard titration solution of sulphuric acid, mL.

**Physical Properties:** Tensile strength (MPa) and elongation at break (%) were measured on ten 0.089 to 0.098 mm by 150 mm dog-bone samples of each material on a universal testing machine according to procedures described in GB/T 1040.1-2006 (Plastics—Determination of tensile properties). The load was applied to failure at a rate of 1 mm/min.

**Film Opacity and Water Vapor Transmission:** Opacity of the CMC composite films was tested by cutting 10 by 40 mm samples and placing them on the inner surface on one side of a cuvette and measuring absorbance at 600 nm on an XP Spectrum 752^#^ ultraviolet spectrophotometer (XP-Spectrum Company, Shanghai, China). Five measurements were made for each sample.

Water vapor transmission rate was assessed under controlled temperature and relative humidity conditions using unit time, unit water vapor pressure difference, and thickness through the unit, and expressed as the unit area of the water vapor volume of the specimen.

The water vapor transmission coefficient of the specimen was calculated according to Equation (3).

(4) $$P=\frac{\Delta m\times d}{A\times t\times \Delta p}$$

where P is the water vapor transmission coefficient of the sample in grams/square centimeter per second Pascal [g cm/(cm^2^·s·Pa)].

Δm is the mass change of the sample in grams (g) during the period t.

A is the sample area through the water vapor in square meters (m^2^).

t is the difference in time between two intervals after the mass change has stabilized in hours (h).

d is the thickness of the specimen in centimeters (cm).

Δp is the difference in water vapor pressure between the two sides of the specimen in Pascals (Pa).

### 2.5. Statistical Analysis

Equality of variance was confirmed using Fisher’s test for raw material chemical analyses, fiber size measurements, and physical properties measurements of CMC composite films. Student’s t test was carried out to compare the samples in pairs at *p* < 0.05. Data were analyzed using SPSS 25.0 statistical package (IBM, Armonk, NY, USA).

## 3. Results and Discussion

### 3.1. Chemical Composition

Cellulose content of eucalyptus bark was 44.9% higher than that of Yunnan pine wood and lower than that of bamboo culms or industrial hemp hurd (Table 1). Hemicellulose content of eucalyptus bark was 26.6% and was higher than that of other three materials. Lignin content of the bark was 27.2% lower than that of Yunnan pine wood and higher than either bamboo culms or industrial hemp hurd. These results were consistent with previous research and indicate that eucalyptus bark-derived cellulose is a suitable alternative [26]. Using this material as a substitute would reduce chemical consumption and allow utilization of a waste product.

### 3.2. Effects of Different Pretreatment Methods on Fiber Yield and Dimensions

Chemical treatment resulted in 100% fiber yield after 10 h of treatment at 70 °C (Table 2). Enzyme treatments produced lower yields. Distilled water treatment resulted in less than 5% fiber yields from bamboo culms and industrial hemp hurd and only 10–20% yield from Yunnan pine wood, despite the 12 h total treatment time. The subsequent use of pectinase alone or in combination with hemicellulase resulted in 90 to 95% fiber yield. Pectin plays important roles in cell wall interactions, especially in primary cell wall formation, and its disruption may facilitate fiber separation. Treatment times for the pectinase treatments were only 6 to 8 h whereas they were 5.5 to 8.5 h for the pectinase and hemicellulase treatments. These results illustrate the potential for producing high fiber yields using enzymatic treatments [27].

The distilled water treatment resulted in little fiber recovery and will not be further discussed. The other treatments had varying effects on the properties of the resulting fibers (Table 3). Pectinase treatment resulted in the shortest Yunnan pine wood fibers whereas the chemical treatment resulted in fibers that were nearly 50% longer. Similarly, pectinase and hemicellulase treated bamboo culms and industrial hemp hurd fibers were only half as long as those from the chemical treatment. Fiber length tended to be greater in all of the chemical treatments compared with the enzymatic treatments, although the pectinase treatment was sometimes similar to the chemical treatment (Table 3). The largest difference in fiber length was found between chemically and enzymatically treated industrial hemp hurd fibers. Decreased fiber lengths may reflect a tendency for enzymatically treated fibers to break more easily as they are separated, which would be detrimental to increasing the tensile strength of any composite. Fiber widths and lumen diameters tended to be similar for the same material regardless of whether the samples were chemically or enzymatically treated. The treatments are less likely to affect fiber width or lumen size, given that their primary effects would be on the cell walls themselves. Cell walls were slightly thicker in pectinase-treated Yunnan pine wood fibers than chemically treated fibers, whereas cell wall thicknesses of pectinase and hemicellulase and chemically treated Yunnan pine wood fibers were similar. Pectin is an important component in primary wood cell wall formation but becomes less important with subsequent lignification. However, the specificity of pectinase for pectin could lead to more efficient separation with reduced breakage.

Fiber length to width ratios can be a useful indicator of potential effects of fiber addition on tensile properties. The addition of fibers with higher length to width ratios may have a greater effect on tensile strength. Length to width ratios tended to be smaller in enzymatically treated fibers than in chemically treated fibers of the same species. As noted earlier, this may reflect a tendency for enzymatically treated fibers to be more brittle and produce shorter fibers, which would reduce tensile properties.

The relative crystallinity of untreated Yunnan pine wood, bamboo culms, and industrial hemp hurd were 38.8, 49.1, and 47.4%, respectively (Table 4). Almost all of the lignin was removed from materials treated chemically or enzymatically and crystallinity was increased. The degree of crystallinity was greatest in Yunnan pine wood but crystallinity also increased in bamboo culms and industrial hemp hurd, although the differences were not significant. Increased crystallinity indicates that chemical and enzymatic treatments removed some amorphous cellulose, resulting in an increase in the proportion of crystalline cellulose. Increased crystallinity may result in stronger reinforcing fibers.

### 3.3. CMC Characterization

**Degree of Substitution:** The substitution degree (DS) on CMC from eucalyptus bark cellulose was 0.89, which is similar to the values obtained for corn stover, straw, and reed CMCs, which ranged from 0.6 to 1.0 [28,29].

**Figure 2:**
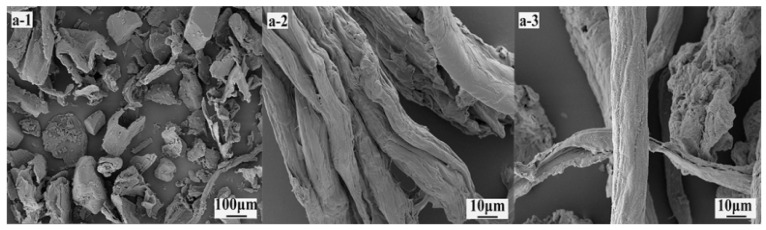
(**a-1**) SEM images showing microstructure differences for unprocessed eucalyptus bark (**a-2**), cellulose microfibrils after chemical treatment, and (**a-3**) CMC produced from the eucalyptus bark cellulose.

**Figure 3:**
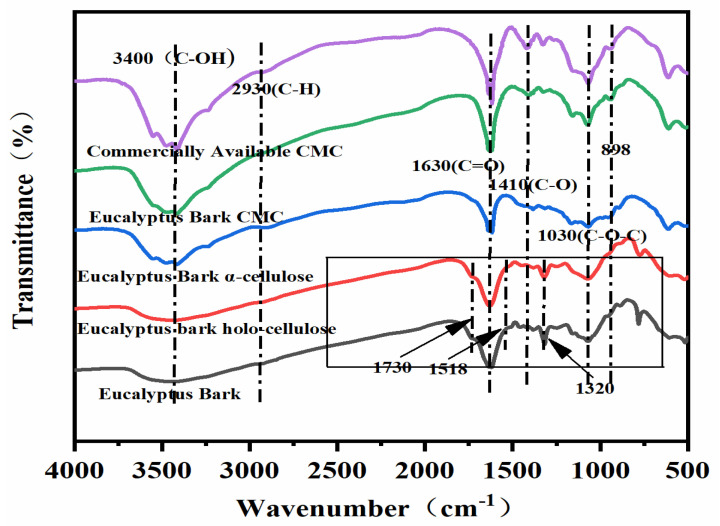
FTIR spectra of bark, holo−cellulose, cellulose, and CMC prepared from eucalyptus bark, as well as commercially available CMC.

**SEM:** SEM examination of eucalyptus bark revealed that it consisted of many substances tightly aggregated together in small granular form (Figure 2a-1). The cellulose recovered from this material was in the form of polymerized fibrous bundles (Figure 2a-2), which became more discrete when the materials were reacted to form CMC with differing degrees of fiber breakage (Figure 2a-3). These changes reflect the effects of alkaline treatment and subsequent esterification, coupled with water penetration into the cellulose bundles, with resulting chain separation.

**FTIR Analysis:** FTIR spectra of commercially available CMC and eucalyptus bark CMC both contained the stretching vibrations of the CMC carboxylate anion COO^-^ at about 1630 cm^−1^, with two characteristic absorption peaks at 1410 cm^−1^ corresponding to the asymmetric (C=O) and symmetric (C-O) stretching vibrations caused by the carboxylic acid group, and an absorption peak at 1030 cm^−1^ corresponding to the stretching vibration of the cellulose C-O-C group. The absorption peak at 898 cm^−1^ is characteristic of the β-glycosidic bond in cellulose [30,31,32,33,34,35,36,37]. No absorption peaks were observed at 1518 and 1320 cm^−1^, which are attributed to the aromatic vibration of the lignin ring and the C-O stretching vibration of the syringyl group, respectively [38], nor was there a peak at 1730 cm^−1^, which is attributed to hemicellulose. These results indicate that cellulose extraction was nearly complete with little evidence of residual lignin or hemicelluloses, and the cellulose was successfully transformed into CMC (Figure 3).

**Figure 4:**
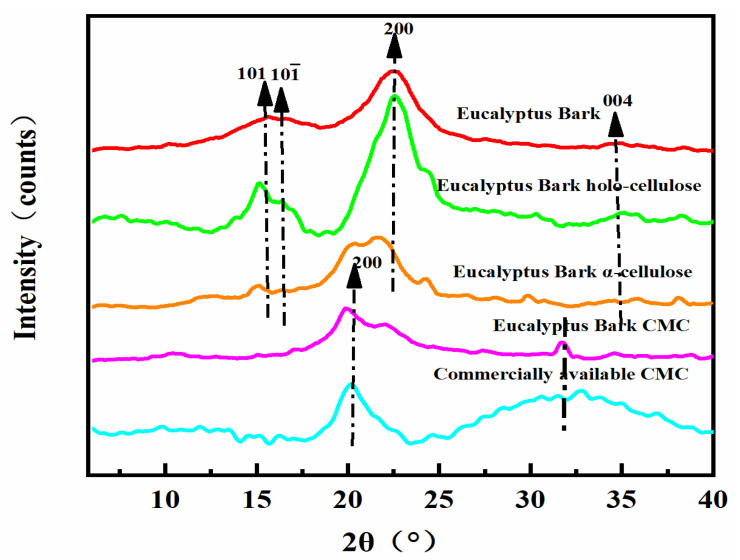
XRD diffractogram showing bark, holo-cellulose, cellulose, and CMC from eucalyptus bark, as well as commercially available CMC.

**X-ray Diffraction**: Eucalyptus bark and holo-cellulose showed diffraction peaks at 2θ = 15.65, 16.45, 22.15, and 34.35°, whereas the cellulose diffraction peaks were at 14.85, 15.4, 21.75, and 34.35°, respectively (Figure 4). All three of the latter peaks correspond to the crystal planes of (101), (10$\bar{1}$), (200), and (004), which are typical reflections of cellulose Type I [39,40,41,42,43]. The relative crystallinity of eucalyptus bark and cellulose were 46.39 and 55.07%, respectively, again indicating that the cellulose was successfully extracted. The diffraction peaks of bark-derived CMC were at 2θ = 19.95 and 31.8°, which were similar to the diffraction peaks obtained from commercially available CMC, indicating successful transformation of cellulose to CMC (Figure 4) [4,43].

### 3.4. Effects of Fiber Treatment Method and Addition of Composite Film Properties

The addition of chemically treated Yunnan pine wood or bamboo culm fibers to the CMC/sodium alginate/glycerol film was associated with decreased tensile strength with increasing fiber content, although the difference in bamboo culm was small. Conversely, tensile strength of the CMC composite film increased with increased industrial hemp hurd fiber. The addition of enzyme-treated Yunnan pine wood, bamboo culms, or industrial hemp hurd fibers enhanced tensile strength of CMC composite films, although this enhancement was small at the lowest addition level (0.1% wt/wt). The results showed that enzymatically treated plant fibers were more likely to produce better CMC composite films (Table 5). Industrial hemp hurd fiber has a smaller wall lumen ratio than Yunnan pine and bamboo fibers. Small compounds such as CMC, sodium alginate, or glycerol are more likely to enter the fiber lumen, especially on enzymatically modified shorter fibers. This should improve tensile strength of the resulting composite.

The addition of chemically or enzymatically treated fibers produced more variable results with elongation at break. Values dropped sharply from the non-modified control but there were no consistent trends associated with fiber additive level or chemical vs. enzymatic pre-treatment. Although fiber addition clearly altered elongation at break, there were no consistent trends with regard to pre-treatment method or additive level (Table 5). Plant fibers usually have high rigidity and should improve tensile strength, but elongation at break could also decrease sharply, as observed in other materials [44].

Opacity is a useful measure for assessing the suitability of a film for commercial purposes. Although opacity varied widely with fiber pre-treatment and additive level, there were no consistent trends with regard to pre-treatment method or concentration. These results suggest that addition of low levels of chemically or enzymatically treated fibers had no consistent effect on opacity, likely because the overall fiber levels remained low.

The background water vapor coefficient for non-modified CMC was 0.20 g·cm/(cm^2^·s·Pa), which is in line with previous reports for CMC films [45]. Addition of 0.1, 0.3, or 0.5 g of chemically or enzymatically recovered fibers to the CMC film had no significant effect on water vapor transmission regardless of plant source (Table 5). These results suggest that these fibers have the potential to improve tensile strength without negatively impacting the ability of the film to function as a water barrier. These attributes would make the films more suitable for food storage.

The data were subjected to an analysis of variance and means were examined using a Tukey’s pairwise comparison test (α = 0.05).

**Figure 5:**
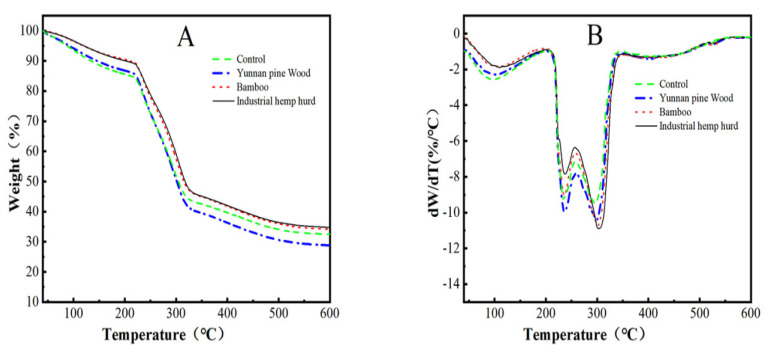
Effect of addition of Yunnan pine wood, bamboo culms, or industrial hemp hurd fibers obtained by pectinase + hemicellulase treatment on (**A**) TG and (**B**) DTG curves of eucalyptus bark CMC composite films.

**Pyrolytic Properties**: The addition of Yunnan pine wood, bamboo culms, or industrial hemp hurd obtained by pectinase and hemicellulase treatment to the CMC was associated with a higher first decomposition peak compared with the non-modified CMC (Table 6). Mass loss at that time was lower for the Yunnan pine wood and bamboo culms but similar for the industrial hemp hurd. The second decomposition peak was slightly lower with the addition of either Yunnan pine wood or bamboo culms fiber but nearly the same as non-amended CMC with addition of industrial hemp hurd. The mass losses at the second decomposition peak were all slightly higher with addition of Yunnan pine wood and industrial hemp hurd losing the most mass. The addition of fibers to the CMC altered both peak temperatures, whereas it decreased mass losses for the first peak and increased them for the second. The final residual weights of the films were 28, 32, 35, and 22 wt%, respectively. The addition of plant fibers resulted in an increase in the required decomposition temperature and an increase in the final weight residue (Figure 5) [4,46].

## 4. Conclusions

Fibers prepared by chemical and enzymatic methods differed in length, width, cell wall thickness, and lumen width, which improved the crystallinity of the fibers. The length:width ratio of the enzymatically prepared fibers was smaller than that of chemically prepared fibers. Wall thickness:lumen width ratios of industrial hemp hurd fibers were the smallest among the three materials. Tensile strength (TS) and elongation at break (EB) of CMC composite films without plant fiber were 26.2 MPa and 7.35%, respectively. TS of CMC composite films was greatly improved by addition of industrial hemp hurd fiber, especially after enzyme treatment. TS of CMC composite films increased to 35.9, 41.3, and 45.2 MPa for chemical, pectinase, and pectinase + hemicellulase treatments, respectively, after adding 0.5 g industrial hemp hurd fiber. EB changed to 1.61, 1.76, and 4.18%, respectively. Addition of modified fibers did not affect opacity or water vapor permeability, indicating that adding low levels of fiber to CMC significantly improved film characteristics making them potentially suitable for food storage.

## Figures and Tables

**Table 1:** Chemical composition of eucalyptus bark, Yunnan pine wood, bamboo culms, and industrial hemp hurd ^a^.

Chemical Compound	Eucalyptus Bark	Yunnan Pine Wood	Bamboo Culms	Industrial Hemp Hurd
Phenyl alcohol extract (%)	2.63 (0.31)	4.79 (0.47)	2.09 (0.53)	3.83 (0.29)
Holo-cellulose (%)	71.5 (0.16)	62.5 (0.15)	76.0 (0.05)	75.5 (0.15)
Celluloses (%)	44.9 (0.20)	42.9 (0.06)	55.9 (0.16)	54.6 (0.19)
Hemicellulose (%)	26.6 (0.12)	19.5 (0.08)	20.1 (0.06)	20.9 (0.25)
Lignin (%)	27.2 (0.05)	32.4 (0.09)	25.2 (0.31)	21.1 (0.06)

^a^ Values represent means of 3 replicates. The numbers in parentheses are one standard deviations.

**Table 2:** Effect of chemical treatment alone or coupled with three sequential enzyme treatments on fiber yield and total treatment time of Yunnan pine wood, bamboo culms, and industrial hemp hurd fibers.

Material	Degree of Fiber Separation (%) and Total Treatment Time (h) ^a^							
	Chemical		Distilled Water		Pectinase		Pectinase and Hemicellulase	
	Fiber Yield (%)	Total Time (h)	Fiber Yield (%)	Total Time (h)	Fiber Yield (%)	Total Time (h)	Fiber Yield (%)	Total Time (h)
Yunnan pine wood	100	10.0	10–20	12.0	90–95	8.0	90–95	8.5
Bamboo culms	100	10.0	<5	12.0	90–95	6.0	90–95	5.5
Industrial hemp hurd	100	10.0	<5	12.0	90–95	8.0	90–95	8.5

^a^ Values represent results from three replicates per material per treatment.

**Table 3:** Effect of chemical or enzymatic treatment on characteristics of fibers derived from Yunnan pine wood, bamboo culms, and industrial hemp hurd ^a^.

Material	Pretreatment Method	Fiber Length (μm)	Fiber Width (μm)	Lumen Width (μm)	Cell Wall Thickness (μm)	Length:Width	Wall Thickness:Lumen Width
**Yunnan pine wood**	**Chemical**	938.6 (463.6–2118.4)	35.4 (20.4–58.5)	20.6 (9.80–48.0)	8.38 (3.61–13.2)	27.4 (13.1–60.4)	0.46 (0.10–0.79)
	**Pectinase**	667.6 (312.7–1537.3)	31.7 (16.7–57.6)	23.6 (9.25–35.4)	12.3 (6.08–23.7)	22.0 (9.94–48.1)	0.57 (0.19–1.06)
	**Pectinase + Hemicellulase**	741.9 (398.5–1507.1)	35.8 (21.1–53.0)	19.6 (9.10–32.0)	9.64 (4.77–17.4)	21.3 (10.3–45.8)	0.53 (0.18–1.15)
**Bamboo culms**	**Chemical**	1096.2 (357.7–2104.1)	18.4 (5.76–40.2)	10.9 (2.77–29.5)	4.13 (1.17–9.11)	69.4 (13.1–195.8)	0.44 (0.09–0.97)
	**Pectinase**	984.6 (344.7–2090.7)	18.5 (9.22–34.4)	9.79 (2.88–22.6)	5.26 (2.17–12.6)	60.0 (19.3–177.4)	0.63 (0.16–1.43)
	**Pectinase + Hemicellulase**	590.9 (293.9–1657.2)	18.8 (7.07–49.1)	10.1 (2.53–37.0)	4.96 (2.03–13.3)	36.7 (9.63–81.9)	0.62 (0.20–1.26)
**Industrial hemp hurd**	**Chemical**	1139.1 (537.3–1963.4)	30.4 (14.2–47.3)	21.6 (5.45–40.4)	3.99 (0.98–11.0)	38.0 (15.9–62.8)	0.19 (0.05–0.56)
	**Pectinase**	514.7 (243.4–797.1)	36.0 (16.6–54.1)	24.9 (7.97–50.9)	4.71 (0.31–11.9)	15.2 (6.85–22.4)	0.21 (0.02–0.53)
	**Pectinase + Hemicellulase**	513.1 (257.7–968.6)	35.5 (13.6–60.5)	21.7 (7.61–45.4)	4.64 (0.17–13.0)	15.2 (7.68–28.4)	0.28 (0.08–0.62)

^a^ Values represent means of 100 fibers per material per treatment while figures in parentheses are the range.

**Table 4:** Effect of chemical or enzymatic treatment on relative crystallinity of Yunnan pine wood, bamboo culms, and industrial hemp hurd fibers ^a^.

Materials	Relative Crystallinity (%)		
	Yunnan Pine Wood	Bamboo Culms	Industrial Hemp Hurd
Chemical treatment	55.1	53.5	55.8
Distilled water treatment	45.1	53.5	51.7
Pectinase treatment	47.1	56.0	54.5
Pectinase + hemicellulase treatment	53.9	50.6	51.7
Raw material	38.8	49.1	47.4

^a^ Values represent means of 3 replicates.

**Table 5:** Effect of addition of chemically or enzymatically derived Yunnan pine wood, bamboo culms, and industrial hemp hurd fibers to CMC film on the tensile strength, elongation at break, opacity, or vapor transmission ^a^.

Material	Pretreatment Method	Plant Fiber (g)	Tensile Strength (MPa)	Elongation at Break (%)	Opacity (A/mm)	Water Vapor Transmission Coefficient (Pv) [g·cm/(cm^2^·s·Pa)]
Control		0	26.2 (0.56) _B_	7.35 (0.62) _A_	7.55 (0.56) _A_	0.20 (0.02) _AB_
**Yunnan pine** **wood**	Chemical	0.1	18.2 (3.68) _BC_	2.87 (1.23) _BCD_	8.06 (1.14) _A_	0.23 (0.03) _AB_
		0.3	15.0 (5.89) _BC_	1.29 (0.27) _CD_	8.17 (0.31) _A_	-
		0.5	11.4 (3.13) _BC_	1.32 (0.22) _CD_	7.63 (0.28) _AB_	-
	Pectinase	0.1	14.4 (3.13) _BC_	0.77 (0.34) _CD_	6.51 (0.46) _AB_	-
		0.3	36.6 (8.51) _AB_	1.11 (0.15) _CD_	7.14 (0.49) _AB_	-
		0.5	38.9 (6.53) _AB_	0.96 (0.10) _CD_	7.18 (0.79) _AB_	0.24 (0.01) _B_
	Pectinase + Hemicellulase	0.1	25.0 (3.71) _BC_	1.06 (0.29) _CD_	7.30 (0.44) _AB_	-
		0.3	25.5 (0.47) _BC_	1.62 (0.03) _CD_	7.51 (0.76) _AB_	-
		0.5	37.4 (4.09) _A_	1.30 (0.30) _CD_	6.75 (0.35) _AB_	0.20 (0.03) _BC_
**Bamboo culms**	Chemical	0.1	26.8 (3.89) _B_	4.32 (0.41) _B_	8.02 (1.02) _AB_	-
		0.3	28.1 (2.50) _AB_	2.22 (0.38) _CD_	6.82 (0.97) _AB_	0.16 (0.03) _AB_
		0.5	23.6 (4.62) _BC_	2.23 (0.55) _CD_	7.80 (1.51) _AB_	-
	Pectinase	0.1	23.3 (5.08) _BC_	1.13 (0.48) _CD_	6.78 (0.36) _AB_	-
		0.3	36.6 (5.14) _AB_	1.64 (0.37) _CD_	7.50 (0.41) _AB_	-
		0.5	45.8 (0.41) _A_	1.34 (0.02) _CD_	7.47 (0.22) _AB_	0.31 (0.02) _A_
	Pectinase + Hemicellulase	0.1	25.3 (4.99) _B_	2.01 (0.64) _CD_	8.11 (0.65) _A_	-
		0.3	29.1 (6.39) _AB_	3.68 (0.53) _BC_	7.97 (1.07) _A_	-
		0.5	38.0 (4.66) _AB_	2.95 (0.61) _BC_	6.63 (0.34) _AB_	0.20 (0.02) _C_
**Industrial hemp hurd**	Chemical	0.1	26.4 (4.30) _B_	3.18 (0.95) _BC_	7.64 (0.03) _AB_	-
		0.3	32.9 (4.93) _AB_	1.09 (0.23) _CD_	7.60 (0.67) _AB_	-
		0.5	35.9 (7.18) _AB_	1.61 (0.28) _CD_	6.09 (2.10) _AB_	0.25 (0.03) _B_
	Pectinase	0.1	21.0 (2.40) _BC_	1.71 (0.30) _CD_	8.55 (0.65) _A_	-
		0.3	27.2 (8.86) _AB_	1.58 (0.34) _CD_	7.67 (0.21) _AB_	-
		0.5	41.3 (3.25) _A_	1.76 (0.28) _CD_	7.73 (0.72) _AB_	0.24 (0.01) _BC_
	Pectinase + Hemicellulase	0.1	23.6 (0.69) _BC_	3.84 (1.48) _BC_	7.57 (0.49) _AB_	-
		0.3	38.9 (4.69) _AB_	4.27 (0.35) _B_	7.24 (0.88) _AB_	-
		0.5	45.2 (3.66) _A_	4.18 (0.43) _B_	6.30 (0.57) _AB_	0.24 (0.04) _B_

^a^ Values represent means of replicates per treatment and figures in parentheses represent one standard deviation. Values followed by the same letter(s) do not differ-significantly from one another by Tukey’s pairwise comparisons (α = 0.05).

**Table 6:** Effect of addition of Yunnan pine wood, bamboo culms, and industrial hemp hurd fibers obtained using pectinase + hemicellulase treatment on decomposition peaks and mass losses of CMC film.

Sample	Decomposition Peak (°C)	Mass Loss (%)	Decomposition Peak (°C)	Mass Loss (%)
Control	231	16	296	52
Yunnan pine wood	236	11	300	57
Bamboo culms	236	11	303	54
Industrial hemp hurd	235	15	295	57

## Data Availability

The data presented in this study are available from the listed authors.
